# Immunosuppressive drugs and fertility

**DOI:** 10.1186/s13023-015-0332-8

**Published:** 2015-10-21

**Authors:** Clara Leroy, Jean-Marc Rigot, Maryse Leroy, Christine Decanter, Kristell Le Mapihan, Anne-Sophie Parent, Anne-Claire Le Guillou, Ibrahim Yakoub-Agha, Sébastien Dharancy, Christian Noel, Marie-Christine Vantyghem

**Affiliations:** Endocrinology and Metabolism, Hôpital Huriez, Lille University Hospital, 59037 Lille Cedex, France; Andrology, Hôpital Calmette, Lille University Hospital, 59037 Lille Cedex, France; Gynaecology –Obstetrics, Hôpital Jeanne de Flandres, Lille University Hospital, 59037 Lille Cedex, France; Endocrine Gynaecology, Hôpital Jeanne de Flandres, Lille University Hospital, 59037 Lille Cedex, France; Hematology, Hôpital Huriez, Lille University Hospital, 59037 Lille Cedex, France; Liver Diseases and Gastroenterology, Hôpital Huriez, Lille University Hospital, 59037 Lille Cedex, France; Nephrology Hôpital Huriez, Lille University Hospital, 59037 Lille Cedex, France; InsermU859 Biotherapies of Diabetes, Lille University Hospital, 59037 Lille Cedex, France

**Keywords:** Fertility, Pregnancy, Transplantation, Auto-immune diseases, Inflammatory diseases, Immunosuppressive drugs: calcineurin inhibitor, Azathioprine, Corticosteroids, Mesalazine, Chloroquine, Cyclophosphamide, Methotrexate, Mycophenolate, Leflunomide, Anti-TNF, mTOR inhibitors, Beta-interferon, Glatiramer, Natalizumab, Fingolimod, Mitoxantrone, Dimethylfumarate

## Abstract

Immunosuppressive drugs are used in the treatment of inflammatory and autoimmune diseases, as well as in transplantation. Frequently prescribed in young people, these treatments may have deleterious effects on fertility, pregnancy outcomes and the unborn child. This review aims to summarize the main gonadal side effects of immunosuppressants, to detail the effects on fertility and pregnancy of each class of drug, and to provide recommendations on the management of patients who are seen prior to starting or who are already receiving immunosuppressive treatment, allowing them in due course to bear children. The recommendations for use are established with a rather low level of proof, which needs to be taken into account in the patient management. Methotrexate, mycophenolate, and le- and teri-flunomide, cyclophosphamide, mitoxanthrone are contraindicated if pregnancy is desired due to their teratogenic effects, as well as gonadotoxic effects in the case of cyclophosphamide. Anti-TNF-alpha and mTOR-inhibitors are to be used cautiously if pregnancy is desired, since experience using these drugs is still relatively scarce. Azathioprine, glucocorticoids, mesalazine, anticalcineurins such as cyclosporine and tacrolimus, ß-interferon, glatiramer-acetate and chloroquine can be used during pregnancy, bearing in mind however that side effects may still occur. Experience is limited concerning natalizumab, fingolimod, dimethyl-fumarate and induction treatments. Conclusion: At the time of prescription, patients must be informed of the possible consequences of immunosuppressants on fertility and of the need for contraception. Pregnancy must be planned and the treatment modified if necessary in a pre-conception time period adapted to the half-life of the drug, imperatively in relation with the prescriber of the immunosuppressive drugs.

## Disease name and definition

Immunosuppressive treatments, with their increasingly varied mechanisms of action, are used in some inflammatory and autoimmune diseases, as well as in transplantation. Frequently prescribed in young people, these therapies can have deleterious effects on fertility, pregnancy outcomes and the unborn child. The aim of this report is to summarise the main gonadal side effects of immunosuppressive drugs, to detail the effects on fertility and pregnancy of each class of drug and to provide practice guidelines on the management of patients who are seen prior to starting or are already receiving immunosuppressive treatment.

## Epidemiology

In Europe, close to 300,000 people have had transplantation, with this number having risen by 45 % since 2000. Autoimmune diseases have become the 3rd leading cause of morbidity and mortality following cardiovascular disease and cancer.

The present review is based on a comprehensive PubMed search between the dates of January 1, 1960, to October 1, 2014, using the search term fertility and pregnancy combined with the different immunosuppressive drugs and discussed according to the multidisciplinary clinical experience of the authors. Most of the published studies, being retrospective, are observational studies without control groups, or are clinical case studies. Randomised, control studies are difficult to conduct for obvious ethical reasons. There is very little data for some drugs, and they are mainly based on animal studies.

Fertility after organ transplantation however has been very well studied, but lacks the ability to distinguish the roles played by immunosuppressive drugs from that of improvement of general health after transplantation. After liver or kidney transplantation, the rates of miscarriage range from 15 to 27 % [1–4], values that are comparable to those of the general population, even if considerably higher incidences (45 %) were observed after kidney transplantation [5] between 1990 and 2003 in the United States (US). The incidences of pre-eclampsia (5 to 15 %), intrauterine growth restriction (IUGR, 9 to 57 %), Caesarean sections (38 to 80 %), prematurity (4 to 50 %) and low-birth weight (32 %), heterogeneous according to the studies and probably according to the underlying maternal condition, seem slightly higher than in the general population [1, 2, 4]. Overall live birth rates, however, currently match that in the US population according to the United States National Transplantation Pregnancy and United Kingdom registries (80 %), as well as Deshpande’s meta-analysis (73.5 %) [4, 6]. The risk of foetal anomalies is very depending on the type of drugs but this risk can now be anticipated.

## Clinical description of the consequences of immunosuppressive drugs on fertility and pregnancy

Most of the time, the intensity of immuno-suppression *decreases with time, especially after the first year in allogeneic organ transplantation*. In addition, allogeneic hematopoietic stem-cell transplant is preceded by the eradication of diseased cells by chemotherapy, using drugs such as cyclophosphamide, a cytotoxic and very gonadotoxic immunosuppressive agent.

### Immunosuppressive drugs and fertility

In transplanted male patients, there is a dose-dependent decrease in plasma concentrations of testosterone, an increase of gonadotrophins and an alteration of spermatogenesis compared to the values of the general population. These gonadal alterations however are less considerable than before the organ transplantation [7–9], as in women [10, 11].

### Immunosuppressive therapies and pregnancy

The efficacy of immuno-suppressive drugs in the treatment of autoimmune diseases or transplants, the emergence of new drugs, and a better knowledge of their side effects, now make pregnancy possible where it was contraindicated several years ago either because of the teratogenic effect of the drugs or because of the underlying maternal condition. Steroids have been involved in increased risk of premature membrane rupture, and ciclosporine in increased prematurity rates but the increased risk of preterm birth in transplant recipient women is also related to the maternal condition and not to immunosuppression. The risk of gestational diabetes and hypertension, however, is amplified by immunosuppressive agents, particularly steroids and tacrolimus. Also, the risk of pre-eclampsia is increased by creatininemia greater than 13 mg/L before pregnancy and/or the use of anticacineurins. In addition, immunosuppressive drugs pose a substantial maternal-foetal infectious risk (bacterial or opportunistic infections especially cytomegalovirus or BK reactivation). The risk of foetal transmission of hepatitis viruses, C in particular, is approximately 5 % and depends on the mother’s viral load after liver transplantation. Moreover, the risk of organ rejection or auto-immune/inflammatory disease reactivation related to the adjustment of immunosuppressive drugs before or during pregnancy is about 2 to 5 % [4]. Finally, breastfeeding should take into account the passage of potential toxic metabolites into the milk and therefore the neonate [12].

Most immunosuppressive agents cross the placental barrier; some are permitted during pregnancy, others are formally contraindicated due to the risk of foetal malformations. The risks of each of these therapeutic classes are detailed in next section.

## Diagnosis

The consequences of immunosuppressive drugs may be anticipated or discovered at any time of a pregnancy in a patient or even years after the birth in the child. The diagnosis of these complications is made upon the medical history (transplantation, rheumatoid arthritis, lupus, bowel inflammatory diseases, multiple sclerosis…) and the analysis of the previous treatment courses both in mother and father before and during the pregnancy. Diagnosis might be clinical by examination of a newborn. Blood renal, haematological, infectious, hormonal and immunologic investigations may me needed. Ultrasound examination is essential especially during pregnancy to screen for teratogenicity

## Differential diagnosis

Immunosuppressive drugs are prescribed for a severe disease and this underlying disease might be responsible by itself for the anomaly. The genetic background may also interfere. Recording all the cases of anomalies recorded with a given treatment may help to understand the mechanisms and avoid further occurrence.

## Aetiologies or effects of the different classes of immunosuppressive agents on fertility and pregnancy

The different drugs are grouped according to their deleterious effects on gametogenesis and on the hypothalamic–pituitary–gonadal axis, followed by potential teratogenic effects of each drug (Table 1).

**Table 1:** Consequences of the main immunosuppressor on fertility and pregnancy

Immunosuppressants	Hypothalamic–pituitary–gonadal axis	Gametogenesis	Mutagenesis	Teratogenesis	Pregnancy	NN	Management
A: Contraindicated drugs when pregnancy is desired							
Methotrexate		M: alteration debated, reversible after stopping for 3 months	M: mutagenic	F: teratogenic without dose effect, especially between 6 and 8 weeks	Increased frequency MC 23%		CI during pregnancy, especially during the first trimester.
		W: No effect: AMH the same at 6 months between treated or non-treated population but less response to ovarian stimulation the first 18 months post-MTX		>30 cases of malformation of the CNS, skull, limbs, IUGR, cardiopathy			Discontinuate at least 3 months before pregnancy in both genders
Mycophenolate		In rats: no effect on fertility	mutagenic *in vivo* in rats	W: clasto-carcino-teratogenic: multiple craniofacial anomalie,…	crosses placenta +++	NN heamato monitoring if data at 2^nd^ or 3^rd^ trimester	Switch to another drug before pregnancy
				M: No effect	increased risk of MC		
Le- and teri-flunomide inhibitor of *de novo* synthesis of pyrimidine	Total elimination of the drug may take 8 to 24 months.	No adverse effect on male or female, even in animals at high doses	neither mutagenic nor clastogenic	Teratogenic in animals: head malformations	insufficient human data	one case of congenital blindness	Stop ≥ 3.5 months before conception or Wash-out with cholestyramine (8gx3/day) or charcoal (50gx4/day) – 10 days to obtain concentration< 0.02 mg/L
				no studies in humans			
							Sperm cryopreservation recommended before treatment in men
Cyclophosphamide cytotoxic alkylating agent	W: FSH/LH increased, even with short exposures	Lasting alteration of ovarian reserve that is dose-, duration- and age-dependent: low AMH	mutagenic	embryolethal and teratogenic without dose effect, especially if early exposure: limbs, dysmorphia, eye,	CI during pregnancy and breastfeeding IUGR	more late exposure, more significant risk NN haemato	Effective contraception to be continued until end of treatment
							Wait for one ovulation cycle after discontinuation before conception
Mitoxantrone	anomalies of the menstrual cycle or even permanent amenorrhea in 7 to 14% of treated patients in correlation with the cumulative dose and the age of exposure	deleterious effect on spermatozoïds and ovocytes leading to fertility alterations. In association with other anti-cancer drugs,	aneuploidism and azoospermia spontaneously improved after 3 to 5 months of treatment discontinuation	teratogenic in animals and humans			Contraindicated in pregnancy .
							A period of 6 months is required after treatment before conception
							Sperm cryopreservation recommended before treatment in men and contraception is required in women.
Thalidomide				teratogenic in humans			
B: Drugs to be used with caution if needed							
mTOR inhibitors	M: inhibitor	M: oligoasthenosper mia, reversible if stopped (debated)	No mutagenic effect *in vitro/in vivo*	embryo- and foetotoxic in animals	−16 reported pregnancies		Substitute with other drug and continue contraception for 12 weeks after stopping treatment
- sirolimus		Testicular atrophy			−3 MC and one child with multiple malformations (+MMF)		No information on breast feeding
- everolimus		W: menstrual disorders 1^st^ year of graft, and 50-60% ovarian cysts, diminishing upon stopping or taking OP in 80% of cases					
temsirolimus							
anti-TNFα	M: no effect	M: no effect	non-mutagenic	W: non-teratogenic in animals and in about 50 pregnancies	1^st^ trimester: Few data etanercept/certolizumab ≠ inflixi/adali:mumab NTR	Crosses placental barrier ± (infliximab until 6 months in NN), less for etanercept, certolizumab	Not recommended to discontinue therapy if desire for pregnancy.
Anti-cytokine	W: no studies			FDA: increased risk cardiac malformations – low level of proof	2^nd^3^rd^ trim: 20 cases: inflixim- adalim- certoliz- umab-: NTR		CI live vaccines
etanercept							
infliximab							
adalimumab							
certolizumab						One child of mother treated entire pregnancy, died from BCG (TB vaccine) complications	Infections without fever
golimumab							
C: Authorised drugs							
Anti-inflammatory	M and W: synthetics and high dose inhibit hypothalamic–pituitary–gonadal axis	promotes apoptosis of germ cells		not more cleft lip-palate	IUGR, premature, eclampsia >	- case of NN ACI after high doses	Possible breast feeding
glucocorticoids		W: In practice, no major impact on fertility			actual role of GC or underlying disease?	- predisposes to unfavourable adult metabolic profile	
Anti-inflammatory		M: oligoasthenoteratospermia reversible after 2 months of stopping; 60% infertility. W: no effect		no effect	increased prematurity	No information on breast feeding	Prescribe folates
Sulfasalazine Mesalazine							Prefer Mesalazine
Anti-metabolites	M and W: no effect	few deleterious effects	mutagenic *in vivo* /*in vitro*	M and W: Non-teratogenic	increased prematurity	Breastfeeding CI ± failure to thrive + reversible haematological NN risk	- Avoid use in a male patient wishing a conception
Azathioprine			carcinogenic and teratogenic in animals	Mutagenic			
							- Discontinuation 3 months before conception
Prodrug of 6 mercaptopurine							
							- US survey of pregnancy if conception by a treated father
							- Sperm cryopreservation recommended
							- Use possible regardless of pregnancy term.
							- Possible breast feeding
Beta interferon	increase of LH	Alterations of ovarian reserve	high molecular weight and should not cross the placenta.	no teratogenic effect	numerous reported pregnancies with either the father or the mother treated : no problem	lower birth weight and higher rate of spontaneous abortions, in the treated mother, even if the treatment is stopped as soon as the pregnancy is known	- Discontinuation not necessary in case of pregnancy
		No sperm alterations					
							- No long term deleterious effects have been reported in the offspring
Glatiramer acetate	increase of LH	Decrease of ovarian surface and number of antral follicles	high molecular weight and should not cross the placenta.	no teratogenic effect	numerous reported pregnancies with either the father or the mother treated : no problem		- Discontinuation not necessary in case of pregnancy
							- No long term deleterious effects reported in the offspring
Chloroquine							- No deleterious effects on pregnancy or breastfeeding
Calcineurin inhibitors	W: no effect	M: asthenoteratospermia if dose > 2mg/kg/day		W: non-teratogenic, limited crossing to placenta (5-20%)	increased infectious risk (CMV)premature / IUGR	passes into breast milk, undetectable in newborns	Use possible during pregnancy; More experience with cyclosporine
Ciclosporine							
Tacrolimus							
				non-teratogenic; 2 cases of congenital malformations	30% premature/ low-birth weight	no renal repercussions in NN	Caution regarding infections
						Reversible involvement of lymphocytes, without clinical repercussions.	Possible breast feeding
						rare cases of transient kidney failure in NN	
D: Drugs with limited experience (biological therapies, inductors)							
Tocilizumab Anti-IL-6 monoclonal Ab	poorly understood effects on fertility clearance: 2 weeks	Large molecule with limited crossing to placenta and breast milk		animal studies: no lethal effect.	poorly understood effects on both fertility and pregnancy	Very few pregnancies reported.	Avoid pregnancy-Limited experience- Continue contraception 3 months after stopping (FDA).
Rituximab anti*-*CD20 monoclonal Ab, B lymphocyte inhibitor	long half-life.	Very limited transplacental crossing (debated)			150 pregnancies, some with early exposure: NTR	Prematurity 15% Haematological anomalies, sometimes infections up to 6–12 months after stopping	Avoid pregnancy less than 12 months after stopping
Abatacept ***(***anti-CD28 Ab or CTLA-4 fusion protein, inhibiting the co-stimulation pathway of the T lymphocytes *(anti-T)*	clearance: 2 ½ months (half-life = 14 days)	-non-altered in animals	large molecule:	non-teratogenic in animals	Several pregnancies with early exposure: NTR		Avoid pregnancy Limited experience
		- W: no studies					
Anankira anti-IL-1 receptor				little teratogenicity in the 1^st^ trimester since limited crossing of placenta ± end of the 2^nd^ trimester when its crossing increases.	<5 pregnancies reported: NTR	Administration in 3^erd^ trimester with increased risk of NN immunosuppression, and CI live vaccines for at least the first 6 months following birth.	Avoid pregnancy Limited experience
Dimethyl fumarate	inhibitor of immune cells and an anti-oxidant	consequences on fertility not known.			consequences on pregnancy not known.		
Natalizumab		Fertility alterations in female but not male animals		No teratogenic effects - effects not fully known yet			Discontinuation of treatment recommended 2 months before conception in men.
Fingolimod or FTY 720 or Sphingosine 1-phosphate receptor modulator		Effects on fertility not well known, − Would have a protective effect on ovarian function at least *in vitro*		teratogenic effects, in animals			Recommended to stop the treatment 2 months before the conception in men and women.
Induction					Unknown effects on both fertility and pregnancy		Avoid pregnancy within 12 months of stopping
- anti-human thymocyte Ig							
*-* IL-2 receptor inhibitors daclizumab							
- belatacept fusion protein (Fc fragment of human IgG1+extracellular CTLA-4							

Note the significant impact of cyclophosphamide on fertility If emergency use needed, start the treatment if possible after the 1^st^ trimester

The website of the French Teratogenic Agent Information Centre [Centre de Référence sur les Agents Tératogènes (CRAT)] (http://www.lecrat.org/) can provide more information

*Ab* antibodies, *CI* contraindicated, *MC* miscarriage, *FDA Food and Drug administration*, *W* women, *M* men, *HAS* French National Authority for Health [*Haute Autorité de Santé*], *ACI* adrenocortical insufficiency, *Ig* immunoglobulin, *IL-2* interleukin-2, *MMF* mycophenolate, *MTX* methotrexate, *NN* neonatal, *OP* oestrogen-progestin contraceptive pills, *NTR* nothing to report, *IUGR* intrauterine growth restriction), *US* United States

*teratogen* substance that causes malformations in the foetus when administered to the mother, *mutagen* substance that increases the number of mutations in the genome, mutations that are likely to promote malformations or an increased carcinogenesis risk, *clastogen* substance likely to induce chromosomal breaks and thus aberrations

### Contraindicated drugs when pregnancy is desired (Table 1)

#### Methotrexate

Study results differ regarding the deleterious effect of methotrexate on *spermatogenesis.* If real, this effect seems to be *reversible after 3 months of treatment discontinuation*. Due to the *mutagenic* risk, men are advised to wait 3 months after stopping treatment to conceive. There is no evidence of a teratogenic effect [13].

The repercussions of methotrexate treatment on female fertility appear to be slight and may even be nonexistent. Serum concentrations of the anti-Müllerian hormone (AMH) were not lower in women treated with methotrexate for rheumatoid arthritis than in controls [14]. The evaluation was done however 6 months after the start of treatment, and the pregnancy rates subsequently obtained were not reported. A poorer response was observed to ovarian stimulation in the 18 months following methotrexate treatment, though it improved thereafter [15].

In contrast, the folic acid antagonist methotrexate has been documented to be teratogenic if administered during the first trimester.of pregnancy*,* even at doses lower than 30 mg/week. Over 30 cases of foetal malformation involving the central nervous system and the limbs were reported in association with IUGR and failure to thrive, etc. [16, 17]. The embryolethal effect of methotrexate is otherwise used in the medical treatment of ectopic pregnancies [16]. The miscarriage rate on treatment is approximately 40 %, considerably higher than that seen in the general population or in those with autoimmune diseases [18]. During the second and third trimester, methotrexate administration is unrelated to a teratogenic effect but could increase the risk of IUGR and low birth weight. Administration apart from conception does not increase the risk of malformations or miscarriage [18]. However a 3-month treatment-free interval between discontinuation of methotrexate and conception is recommended.

#### Mycophenolate (purine synthesis inhibitor)

Mycophenolate, being non-diabetogenic, is one of the most commonly used immunosuppressive drugs in transplantation.

There is no data on the effects of mycophenolate on male fertility. The 205 pregnancies involving 152 transplanted fathers who had been treated with mycophenolate were associated with a similar risk of *prematurity* (10 %) and *malformations* (3 %) as in the general population [19].

The AMH levels of female patients treated with mycophenolate for lupus were not lower than in a control population [20]. A very considerable amount of mycophenolate crosses the placental barrier. In rats, there is a *teratogenic* and mutagenic effect. In women, mycophenolate poses an *increased risk of miscarriage* (32 % to 45 %) and *multiple craniofacial congenital malformations* (MMF-associated embryopathy (EMFO tetrada: Ear, Mouth, Fingers, Ocular/Organ malformation) in 26 % of cases after first trimester exposure to MMF according to The European Network of Teratology Information Services [21]. Foetal toxicity is present throughout the first trimester and seems to be cumulative depending on the clinical cases reported. For this reason, *treatment must imperatively be modified in the event that pregnancy occurs, and the prescription of mycophenolate should be avoided in young, transplanted women with a potential desire to become pregnant.* There are few data on the exposure to mycophenolate during the second part of pregnancy, but the treatment could result in blood count abnormalities in exposed newborns. The U.S. Food and Drug Administration (FDA) launched a systematic information programme for patients on the teratogenic risk including the issuance of a written information document, signature of informed consent before the prescription, and incentive to participate in a registry of pregnancies occurring on mycophenolate or within 6 weeks of its discontinuation.

#### Leflunomide and teriflunomide

In animal models, *teriflunomide*, an inhibitor of *de novo* synthesis of pyrimidine, active metabolite of leflunomide does *not have adverse effects on male or female fertility*, but both drugs are *embryotoxic and teratogenic,* promoting the development of malformations of the axial skeleton and head (microphthalmia, hydrocephaly). There is little data from pregnant women, but cases of complex morphological-functional abnormalities (congenital blindness and perceptive deafness) were reported in children with parents exposed to leflunomide in the pre-conception period or during pregnancy [22, 23]. *These drugs are therefore contraindicated in the pre-conception period (the 3½ months before pregnancy) and during pregnancy. A washout procedure may be proposed with cholestyramine (8 g, 3 times daily) or activated charcoal (50 g, 4 times daily) for 10 days in order to shorten the time period needed between discontinuation of the treatment and conception,* since the total elimination of the drug may take 8 to 24 months. A sperm cryopreservation is recommended before treatment.

#### Cyclophosphamide

Cyclophosphamide, a cytotoxic alkylating agent widely used in allogeneic bone marrow transplantation, permanently alters the ovarian reserve in a manner that is dose-, duration- and age-dependent. This has been well demonstrated through decreased plasma concentrations of AMH and increases in gonadotrophins, with some variations correlated to the cumulative dose of cyclophosphamide, even for short exposures [19, 24].

Cyclophosphamide in animals is *mutagenic, teratogenic and embryolethal*. In humans, it is a confirmed teratogenic agent that promotes IUGR, malformation of the extremities and the head (ocular involvement, facial dysmorphia, craniostenosis, hydro- or microcephaly). The reported cases are rare so that it is difficult to establish a dose–response relationship; some malformations have been observed at low doses, while newborns that had had high dosage exposures were born healthy. The malformations seem to be secondary to exposure during the 1st trimester. However, the *haematological side effects observed in the neonatal period* are all the more frequent the later the exposure during the 2nd and 3rd trimesters of pregnancy. *The teratogenicity of cyclophosphamide absolutely contraindicates its use in pregnancy. If its use is essential (e.g., breast cancer diagnosed during pregnancy), the treatment will be prescribed after the 1st trimester if possible. Conception is theoretically possible about two days after the end of the treatment. In practice, effective contraception must be continued until the end of treatment, and a minimum of one ovulation cycle after the end of treatment is recommended before considering conception.*

#### Mitoxantrone

Mitoxantrone (Table 1), sometimes used in multiple sclerosis, also a chimiotherapy in leukemia and prostate cancer, induces anomalies of the menstrual cycle or even permanent amenorrhea in 7 to 14 % of treated patients The effects are correlated with the cumulative dose and the age of exposure [25, 26]. Mitoxantrone has a *deleterious effect on spermatozoïds and ovocytes leading to fertility alterations*. In association with other anti-cancer drugs, mitoxanthrone may be responsible for aneuploidism and azoospermia spontaneously improved after 3 to 5 months of treatment discontinuation [27, 28]. This drug is *teratogenic* in animals and humans and is therefore *contraindicated in pregnancy. Sperm cryopreservation is recommended* before treatment initiation in men and contraception is recommended as well in women [29]. A period of 6 months is required after treatment discontinuation before conception.

In conclusion, these 5 immunosuppressive agents are teratogens, and other immunosuppressive agents must be used when pregnancy is desired. In addition, cyclophosphamide alters the ovarian reserve, thus warranting a consultation in a specialised centre for the purpose of ovarian preservation before prescription of the drug.

### Drugs to be used with caution (Table 1)

#### Anti-TNF-α (tumour necrosis factor alpha) (anti-cytokine)

The most commonly used anti-TNF alpha drugs are first, etanercept, a TNF inhibitor acting as a soluble receptor, with a half-life of 72 h; and second, the anti-TNF antibodies (infliximab, adalimumab, certolizumab, golimumab), with a half-life up to 14 days.

Various andrological studies in men are not supportive of spermatic alteration secondary to treatment with TNF-alpha inhibitors. Therefore there are no recommendations to stop treatment if conception is desired [30]. Compared to case controls in spondyloarthritis, the levels of inhibin B, testosterone and gonadotrophins are unchanged by these drugs [30, 31], particularly etanercept and adalimumab.

There have been no studies in women on the effects of anti-TNF alpha agents on the hypothalamic–pituitary–gonadal axis and the ovarian reserve.

Anti-TNF alpha agents are not teratogenic in animals, nor mutagenic in pre-clinical tests.

These drugs cross the placenta − infliximab in particular, which has been detected up to 6 months after childbirth in the child’s blood [32]. There are more reported cases of exposure to infliximab and adalimumab in the first trimester of pregnancy than to etanercept or certolizumab. The reported cases of use of these drugs in the second part of pregnancy are extremely rare. The data however are reassuring and do not show malformations in children born from these pregnancies [33]. A risk of VACTERL association (anomalies of the vertebrae and/or limbs, anal atresia, tracheoesophageal fistula, renal malformation) and heart defects have been noted by the Food and Drug Administration (FDA), but this report has methodological biases [34].

The frequency of infections however, including reactivation of tuberculosis in the mother, is higher, particularly with certozilumab [35]. A case of severe tuberculosis that resulted in death was reported after BCG vaccination in one child.

*In practical terms, considering the still limited experience available as to the use of these drugs, it is recommended that contraception be continued in women receiving TNF-alpha inhibitors, prolonged for 3 weeks after stopping etanercept and 3 months after stopping anti-TNF-alpha antibodies despite the lack of demonstration of teratogenic effects. In addition, infections may not be accompanied by fever in patients using anti-TNF alpha drugs. If the use of anti-TNF alpha treatment is necessary, it is recommended that the last injection be given at the start of the 3rd trimester of pregnancy due to the long half-life of elimination. At birth, the child is immunocompromised, and this may continue up until 6 months following the last maternal injection, making the postponement of live vaccinations mandatory (2013 HAS [French National Authority for Health] Recommendations).*

#### mTOR inhibitors: sirolimus, everolimus, temsirolimus

In addition to their immunosuppressive properties, the mTOR inhibitors are also used as anti-tumour agents, notably in pancreatic neuroendocrine tumours.

Sirolimus alters the functioning of the *hypothalamic–pituitary–gonadal axis* [36, 37]. As a result, men with kidney transplants who are treated with sirolimus have lower serum testosterone levels and higher plasma concentrations of gonadotrophins than on other immunosuppressive drugs. These values nevertheless remain within normal limits. These variations are positively correlated to the dose of sirolimus and are independent of other variables, such as graft function, age of the patient, body mass index, duration of dialysis, existence of post-transplantation diabetes and use of steroids [38]. Similarly, high levels of everolimus (combined with corticosteroids and calcineurin inhibitors) are the cause of poorer recovery of testosterone, FSH and LH levels after transplantation [39]. Sirolimus qualitatively and quantitatively alters *spermatogenesis* in men, as observed in 132 heart transplant patients [40–42]. This adverse effect is purportedly reversible upon stopping the treatment [41, 43], although this reversibility is controversial [42]. A testicular histological study done in a patient treated with sirolimus who needed surgical intervention for leydigioma showed testicular atrophy and vacuolation of the seminiferous epithelium [44], in agreement with the animal studies.

Young women treated with sirolimus and tacrolimus have an increased risk of oligospanio- or amenorrhoea) and ovarian cysts (50 to 62 % of cases) in the first year of the transplant [45–47]. These cysts diminish in 80 % of cases when sirolimus is stopped or with the use of combined oestrogen plus progestin contraceptive pills. Some cysts, such as mucinous cystadenomas or haemorrhagic cysts, require surgical cystectomy [47]. Their occurrence is allegedly promoted by insulin resistance induced by immunosuppressive agents, but this hypothesis has not been demonstrated.

In animal models (rats), treatment with tacrolimus, sirolimus or a combination of both lead to a decrease in the ovarian surface, the number of ovulation cycles, uterine size, and aromatase expression, although with the corpora lutea present. Conversely, sirolimus treatment allegedly protects the ovarian reserve and prevents the transition of pre-antral follicles to antral follicles by modulating the mTOR and signalling pathways [48].

In animals, sirolimus does not have an *in vitro* or *in vivo mutagenic* effect, but it is *embryo- and foetotoxic*. In the clinical setting, *sixteen pregnancies* were reported between 2004 and 2012 while using sirolimus or everolimus [49]; of these, 3 resulted in miscarriage and one child had many malformations but had received mycophenolate in early pregnancy. *Currently, experience with the use of this treatment during pregnancy is insufficient. Even though the published data seem to be reassuring, it is recommended that another drug be used as a substitute for mTOR inhibitors, and that contraception be continued for 12 weeks after stopping treatment due to the clearance time.*

Finally, these 2 therapeutic classes have little effect on fertility and pregnancy, but the available experience of use is still limited (about 10 years), and as a result their prescription must be avoided when possible.

### Treatments associated with a low risk of deleterious effects on fertility and pregnancy (Table 1)

#### Glucocorticoids

The use of glucocorticoids has consequences on fertility [50]. Therefore, synthetic (and even endogenous) corticosteroids have an *inhibitor effect on the male hypothalamic–pituitary–gonadal axis*: a) through direct and indirect action on the level of transcription of the GnRH receptor gene on the pituitary cells; b) by modulating the expression of the LH receptor genes or of testosterone biosynthesis enzymes, whether through genomic effects or not; and c) through inhibition of the adrenal synthesis of androgens. *In the ovaries*, low doses of corticosteroids play a positive role in steroidogenesis, the maturation of oocytes, ovulation and maintenance of the corpus luteum. However high-dose exogenous intake is often deleterious.

*In vitro*, glucocorticoids exert a proapoptotic effect on the germ cells of rat testes and ovaries of the female foetus [51]. In practical terms however, *there are no major consequences on the fertilisation capacities in women* receiving corticosteroid treatment [52].

The incidence of *IUGR, low-birth weight, pre-eclampsia, hypertension, diabetes and prematurity is increased* in cases of exposure to corticosteroids during pregnancy compared to the general population, regardless of the dosage and the type of corticosteroid use [53–55]. In addition, high doses of glucocorticoids during intrauterine life risk may cause *neonatal adrenal insufficiency, but also* dysfunction of the hypothalamic–pituitary–adrenal axis and *foetal “programming”,* thus predisposing to an unfavourable metabolic profile as an adult [56].

#### Sulfasalazine

The use of sulfasalazine is the cause of *oligo-astheno-teratospermia and infertility* in over 60 % of treated men [57], although it is reversible after two months after stopping treatment. However, the hypothalamic–pituitary–gonadal axis changes little, as the plasma concentrations of prolactin, testosterone and gonadotrophins are normal [58]. *In women, no effects* of this treatment have been reported on fertility parameters. There has been no demonstration of congenital foetal anomalies on this therapy [59], but *the prescription of folate supplementation is advised due to its mechanism of action.*

#### Mesalazine

The hypothalamic–pituitary–gonadal axis and the spermatic parameters are not altered with treatment using this drug [60]. In fact, replacement of sulfasalazine by mesalazine improves the spermatic parameters [58]. There has *been no demonstration of congenital foetal anomalies or maternal-foetal complications other than prematurity,* which was slightly increased with mesalazine [61].

#### Azathioprine (purine analog, precursor of 6- mercaptopurine)

Azathioprine is mutagenic. The rare andrological studies are not supportive of alterations of fertility [62]. More than 1000 pregnancies issued from men treated with azathioprine or 6-mercaptopurine have been published and no teratogenic effects have been reported. Nevertheless, chromosomal anomalies have been described on spermatozoids of male subjects during the treatment and the year following its discontinuation.

There is little published data in women, but azathioprine does *not seem to have detrimental effects on fertility*. The AMH concentrations of patients treated with azathioprine, mycophenolate or calcineurin inhibitors for lupus were not lower than in the control population [19].

Azathioprine is *teratogenic* in animals, as treated pregnant females and their foetuses have presented with chromosomal abnormalities on the circulating lymphocytes.

A*zathioprine is authorised during pregnancy*. Although it crosses the placental barrier, this agent cannot be converted to an active metabolite, as the foetal liver lacks the specific enzyme. No teratogenic risk has been demonstrated in the many data, both in women and in men [61, 63–65]. In contrast, prematurity [61], some cases of immunosuppression that quickly resolve [66] and/or reversible involvement of the blood cell lineages have been observed in newborns. Otherwise, *breastfeeding is not contraindicated,* since the active metabolite, 6 mercapto-purine, rarely passes, and if passes, remains at very low level, into breast milk [67].

*If azathioprine is required for control of the inflammatory disease or the graft, it may be used during pregnancy, but an ultrasound monitoring should be proposed. Amniocentesis may be discussed but can only discard usual caryotypic alterations. A sperm cryopreservation is recommended before instauration of the treatment in a male subject because of the mutagenic effect. If a treated male subject wishes a conception, the treatment should be stopped if possible at least 3 months before conception (a cycle of spermatogenesis).*

#### Ciclosporine (calcineurin inhibitor)

*No significant difference in the hormonal parameters* (FSH, LH, testosterone and prolactin) were observed between kidney transplants treated with ciclosporine and those treated with tacrolimus [68]. However, rats treated with ciclosporine had irreversible testicular damage [7]. In men, doses greater than 2 mg/kg/day [9] have been implicated in *asthenoteratospermia*. No significant difference was seen in men receiving lower doses compared to the control group. Therefore it is not recommended that treatment be discontinued if pregnancy is desired. Plasma concentrations of AMH in patients treated with calcineurin inhibitors for lupus were not lower than in a control population [20].

Limited amounts of ciclosporine cross the placenta (5–20 %), and it does *not result in teratogenic effects* [69].

No renal repercussions attributable to ciclosporine were observed during the follow-up of several hundred children exposed in utero. *Reversible involvement of B or T lymphocytes* without clinical consequences, prematurity, and IUGR have sometimes been reported. These neonatal effects may be attributed to ciclosporine but also to the disease and/or associated treatments.

#### Tacrolimus (calcineurin inhibitor)

There are a large number of published data on pregnant women exposed to tacrolimus, and they show no evidence of an increase in malformations [70]. The experience acquired over 13 years [71] in 37 women treated with tacrolimus after liver transplantation and who gave birth to 49 children resulted in:

- 30 % premature births [72]
- 32 % birth weight below the 50th percentile
- 5 to 6 % congenital malformations [70]
- rare cases of transient hyperkalaemia and kidney failure in the child

#### Beta interferon and glatiramer acetate

Studies are scarce but significant alterations of ovarian reserve have been reported both with *beta interferon and glatiramer acetate* used in the treatment of multilocular sclerosis [73]. These 2 molecules have a high molecular weight and should not cross the placenta. Neither spermatic alterations nor teratogenic effects have been observed in the numerous reported pregnancies with either the father or the mother treated with these 2 molecules [74, 75]. Interferon is however associated with a lower birth weight and a higher rate of spontaneous abortions, in the treated mother, even if the treatment is stopped as soon as the pregnancy is known [76, 77]. The discontinuation of these treatments 3 months before conception is recommended each time the disease is not a frequent relapsing form. In other cases, the treatment might be stopped as soon as the conception is proven. If necessary, the treatment might be pursued during the whole pregnancy. No long-term deleterious effects have been reported in the offspring [78–81].

#### Chloroquine

Chloroquine appears to have a *deleterious effect on sperm quality in vitro* and *in vivo* in animal studies. Little data are available in males, unlike in pregnant women in which there is a large amount of reassuring data. The teratogenic risk related to the use of hydroxychloroquine is very low [82].

*Chloroquine may be used at prophylactic doses for malaria. In other situations, if continuation of the therapy is required for good control of the treated disease, the lowest possible dose should be used during pregnancy.*

In conclusion, if these drugs are required for control of the inflammatory disease or maintenance of the graft, they may be used during pregnancy.

### Recent drugs in which the effects are not fully known (Table 1)

#### Biological therapies

Biological therapies (tocilizumab, rituximab, abatacept, anankira) seem to have few teratogenic effects in the first trimester, since a very limited amount cross the placental barrier. This is in contrast to the end of the 2nd trimester, in which crossing is increased [83–85]. If administered in the 3rd trimester, some of these drugs carry an increased risk of neonatal immunosuppression, and their use contraindicates the use of live vaccines for at least the first 6 months after birth. Abatacept and anankira appear to be associated to a lesser extent with side effects than the anti-TNF alpha agents, infliximab in particular [34, 86].

Very few pregnancies have been reported. Due to this scarcity of experience, *the prescription of biological therapies is discouraged if pregnancy is desired, both in women and men due to the still poorly understood long-term side effects* [87]*.* Continued use of contraception is advised for 3 months following discontinuation of these treatments due to their long half-life.

#### Immunomodulators in multiple sclerosis

Besides *beta interferon and glatiramer acetate* use (Table 1), and mitoxantrone (Table 1), *dimethyl fumarate* acts as an inhibitor of immune cells and an anti-oxidant. Its consequences on fertility and pregnancy are not known.

*Natalizumab* alters fertility in female, but not male, animals. No teratogenic effects have been observed in women but the effects are not fully known yet [88, 89]. The discontinuation of treatment is recommended 2 months before conception in men.

The effects of *fingolimod* on fertility are not well known, but fingolimod seems to have a protective effect on ovarian function at least *in vitro* despite its teratogenic effects in animals [90, 91]. Therefore, it is recommended to stop the treatment 2 months before the conception in men as well as in women [92].

Except for *beta interferon and glatiramer acetate, which might be prescribed if necessary, the other immunomodulators used in multiple sclerosis should be stopped two (natalizumab, fingolimod) to six months* (*mitoxanthrone*) *before conception,* with a possible wash-out procedure for *teriflunomide*.

#### Induction immunosuppressive treatments

The effects of induction treatments (rabbit anti-human thymocyte immunoglobulins; interleukin-2 receptor antagonists such as daclizumab; belatacept) on fertility and pregnancy have not been studied.

## Management of patients using immunosuppressive drugs

The management prior to the patient starting treatment with an immunosuppressive agent, or when the patient is already receiving this immunosuppressor treatment is summarized in Tables 1 and 2 [93–95].

**Table 2:** Management of patients prior to starting or already taking immunosuppressive drugs

A: Management prior to starting immunosuppressive treatment
1. Patient information to clarify the consequences of the planned treatment on future fertility and the couple desire for subsequent fertility (”Bioethics Law of 2004 and 2011 France (Art. L. 2141-)11)
2. Verify the absence of pregnancy.
3. Send to a fertility preservation centre, particularly if cyclophosphamide (see Table 3).
4. Effective contraception until desire for pregnancy, even in case of supposedly already altered ovarian reserve if the treatment is teratogenic and/or pregnancy would be at risk due to the underlying disorder. The metabolic effects of calcineurins and mTOR inhibitors as well as glucocorticoids will require the prescription of a progestin-only rather than a combined oestrogen-progestin contraceptive.
5. Screening for potential gynaecological neoplasias (cervical-vaginal smear, mammography) that could be worsened by immunosuppressive treatment
B Pre-conception management of a parent treated with immunosuppressive agent
1. Pre-conception consultation to discuss stopping/changing immunosuppressive drug with referring team.
2. Preconception consultation with the couple: an explanation will be offered concerning the possible strategies and obligations in order to minimise the maternal-foetal risks if pregnancy is possible; these include the need for a stable disease, multidisciplinary management, a possible period of weaning from the treatment or immuno-suppression switch, the risk of organ rejection and/ or reactivation of an underlying disease, the risk of transmitting a genetic disorder causing organ failure (Wilson’s disease, polycystic kidney disease, etc.).
3. The pregnancy will need to be planned, and the treatment will need to be modified if necessary in a pre-conception time period adapted to its half-life (See Table 1, last column).
4. If pregnancy inadvertently occurs while taking immunosuppressive treatment, reassure the patient, test for known complications (ultrasound fetal examination) and consider stopping or replacing the treatment according to the context.
5. During a planned pregnancy while on authorised treatment, the dosages of immunosuppressive agents should be closely monitored in order to avoid overdose situations that are shared with the foetus, but also to avoid underdosing, which would compromise the control of the disease or the function of the graft. During pregnancy there are variations in absorption and metabolism of the drugs.

Pregnancy, when an option, must be planned under conditions of strict monitoring, when the disease is not in an acute phase. These findings prompt the recommendation to delay pregnancy up to l to 2 years after the transplant, when the immunosuppressive doses will be lower and the menstrual cycles will have spontaneously re-established [14].

Most immunosuppressants cross the placental barrier and, depending on the foetal malformation risk, some are authorised during pregnancy (azathioprine, corticosteroids, anticalcineurins, ß interferon, glatiramer acetate, chloroquine), and others are formally contraindicated (methotrexate, mycophenolate, le- and teri-flunomide, cyclophosphamide, mitoxantrone). If the latter treatments (cyclophosphamide in particular) need to be prescribed for emergency purposes, it is preferable that they be started after the 1st trimester of pregnancy. For some drugs that have fewer data but seem reassuring, the use may be continued during the pregnancy if needed (anti-TNF alpha, mTOR inhibitors).

The preconception time period for stopping varies with each drug and depends on its clearance time (5 half-lives): 12 weeks for the m-TOR inhibitors; 4 weeks to 2 years for le- and teri-flunomide (according to whether or not the wash-out protocol with charcoal and cholestyramine is used); 12 weeks in men and in women for methotrexate; one ovulation cycle for cyclophosphamide; and 6 weeks for mycophenolate.

## Prognosis and unresolved questions

In the 21st century, immunosuppressive drugs are indicated in many different diseases due to their efficacy but also due to a better understanding of their mechanisms of action and their side effects. This efficacy has enabled improvement of underlying diseases, making parenthood an option in some young patients who previously had definitive life-threatening conditions. Nevertheless, these treatments remain powerful therapies that require skilful handling; therefore it should be borne in mind that the long-term consequences are still not fully understood. As an example, the marketing authorisation for mTOR inhibitors only dates back 10–15 years. Although childbirth is possible with these treatments, this undertaking is never without risk to the mother and child. It must be anticipated very far in advance, before the prescription of the immunosuppressants, in order to preserve fertility, and then before conception in order to adjust the immunosuppressive treatment. Close monitoring of the pregnancy in conjunction with the prescriber of the immunosuppression and long-term follow-up of children from these pregnancies is recommended. The potential consequences of immunosuppressive drugs in children who have received this exposure during pregnancy must still be evaluated once they are adults. Recommendations for use are established with a rather low level of proof, which needs to be taken into account when informing and monitoring patients. This multidisciplinary management for planned parenthood must be discussed with the couple so that they understand and accept the challenges and obligations.

## Abbreviations

Ab: Antibodies
CI: Contraindicated
MC: Miscarriage
FDA: Food and Drug administration
HAS: French National Authority for Health [*Haute Autorité de Santé*]
ACI: Adrenocortical insufficiency
Ig: Immunoglobulin
IL-2: Interleukin-2
IUGR: Intrauterine growth restriction
MMF: Mycophenolate
MTX: Methotrexate
NN: Neonatal
OP: Oestrogen-progestin contraceptive pills
NTR: Nothing to report
W: Women
M: Men
